# Comparison of Performance of Equations for Estimated Glomerular Filtration Rate in Chinese Patients with Biopsy-Proven Diabetic Nephropathy

**DOI:** 10.1155/2019/4354061

**Published:** 2019-09-15

**Authors:** Yiting Wang, Junlin Zhang, Geer Teng, Yucheng Wu, Qianqian Han, Hanyu Li, Tingli Wang, Fang Liu

**Affiliations:** ^1^Division of Nephrology, West China Hospital of Sichuan University, Chengdu 610041, China; ^2^The Faculty of Social Development and Western China Development Studies, Sichuan University, China

## Abstract

**Background:**

The performance of various equations for estimated glomerular filtration rate (eGFR) in patients with diabetes remains controversial. We aimed to evaluate the performance of equations for eGFR in Chinese patients with diabetic nephropathy (DN).

**Methods:**

This is a retrospective study included in 308 patients with type 2 diabetes and biopsy-proven DN who were followed up at least one year. eGFR was calculated using chronic kidney disease epidemiology (CKD-EPI) equations based on serum creatinine (eGFR_CKD-EPI-Cr_), cystatin C (eGFR_CKD-EPI-CysC_), and joint equations (eGFR_CKD-EPI-Cr-CysC_), respectively. End-stage kidney disease was defined by initiation of renal replacement therapy. The eGFR concordance between equations was assessed by Bland-Altman plots. Log-rank and multivariable logistic regression were employed to evaluate the performance of equations.

**Results:**

Overall, the proportion of patients with eGFR < 60 mL/min/1.73m^2^ was 53%, 70%, and 61% by the equations of eGFR_CKD-EPI-Cr_, eGFR_CKD-EPI-CysC_, and eGFR_CKD-EPI-Cr-CysC_, respectively. Higher disconcordance was observed between equations when eGFR > 60 mL/min/1.73m^2^. Compared with eGFR_CKD-EPI-Cr_, 39% of patients were reclassified (reclassified group) from CKD 1-2 stages to CKD 3-5 stages by eGFR_CKD-EPI-CysC_ and they presented significantly longer diabetic duration, heavier proteinuria, advanced pathological lesions, and poorer kidney outcomes. Multivariable logistic regression indicated cystatin C was independently associated with advanced glomerular classifications.

**Conclusion:**

eGFR equations incorporating cystatin C are superior to eGFR based on creatinine alone for detecting kidney injury in the early stage. The independent association between cystatin C and glomerular classifications might contribute to it.

## 1. Introduction

The past few decades have witnessed a marked increasing prevalence of type 2 diabetes, especially in China, and the global prevalence of microvascular and macrovascular complications associated with diabetes increases dramatically [1, 2]. Diabetic nephropathy (DN) has become the leading cause of end-stage kidney disease (ESKD) worldwide [3, 4]. The utilization of renin-angiotensin-aldosterone system blockers and improvements in glycemic, blood pressure, and lipid control slow the progression of chronic kidney disease (CKD) to a degree [5]. Indeed, glomerular filtration rate (GFR) guides the clinical management of CKD and is an independent predictor of kidney injury, all-cause/cardiovascular mortality, and kidney failure [6]. Therefore, accurate estimation of GFR to identify CKD and predict kidney outcome is highlighted.

The Kidney Disease Improving Global Outcomes (KDIGO) 2012 Clinical Practice Guideline for the Evaluation and Management of CKD [7] recommends initial use of 2009 CKD-Epidemiology Collaboration equation based on serum creatinine (eGFR_CKD-EPI-Cr_) instead of the Modification of Diet in Renal Disease equation. They also suggest use of the 2012 CKD-EPI equations (eGFR_CKD-EPI-CysC_, eGFR_CKD-EPI-Cr-CysC_) to confirm kidney function when cystatin C has been measured, particularly for patients with eGFR_Cr_ of 45–59 mL/min/1.73 m^2^ who do not have markers of kidney damage. However, the performance of various equations in CKD cohorts remains controversial due to serum creatinine is influenced by age, muscle mass, sex, and race; cystatin C level is affected by ages, body mass index, diabetes, and inflammation. Patients with DN are recognized as a special community in CKD. Also, the implications and predictive potential of different equations in patients with DN have yet to be elucidated. The objective of the study was to compare the performance for detecting kidney injury of equations of eGFR_CKD-EPI-Cr_, eGFR_CKD-EPI-CysC_, and eGFR_CKD-EPI-Cr-CysC_ in patients with DN in a single center in Southwest China.

## 2. Method

### 2.1. Study Population

This is a retrospective cohort study. From November 2003 to March 2018, a total of 308 Chinese patients with type 2 diabetes mellitus (T2DM) and biopsy-proven DN in West China Hospital of Sichuan University were recruited and followed up for at least one year by routine clinical visits. Patients with T2DM and proteinuria > 0.5 g/24 h or eGFR decline were indicated to receive kidney biopsy in our hospital. The diagnoses of T2DM and DN were based upon the criteria recommended by the American Diabetes Association (ADA) in 2018 [8] and the Renal Pathology Society in 2010 [9]. The ESKD was defined by initiation of renal replacement therapy (hemodialysis, peritoneal dialysis, or kidney transplantation). Patients who had malignances, nondiabetic renal disease (NDRD), and NDRD+DN were excluded from the study.

The protocol of study was approved by the ethics committee of West China Hospital of Sichuan University and conducted based on the principles of the Declaration of Helsinki; written informed consents were obtained at the time of biopsy from all the patients.

### 2.2. Clinical and Pathological Characteristics

Diabetic history, data including physical examinations (body mass index, blood pressure, and examination of diabetic retinopathy) and laboratory tests (HbA1c, 24-hour protein excretion, serum creatinine, serum cystatin C, serum lipid), were collected at the time of kidney biopsy from the hospital information system. Creatinine was measured using Jaffe's assay. Serum creatinine value was calibrated to isotope dilution mass spectrometry (IDMS). Serum cystatin C was measured using an automated particle-enhanced immunoturbidimetric method. Blood samples were collected after 12 hours of fasting in all the patients [10]. Pathological lesions were routinely assessed under light and electron microscopy by at least two nephropathologists according to criteria proposed by the Renal Pathology Society in 2010 [9].

### 2.3. Statistical Analysis

GFR was estimated using the equations [11] of eGFR_CKD-EPI-Cr_, eGFR_CKD-EPI-CysC_, and eGFR_CKD-EPI-Cr-CysC_, respectively. The CKD stages 1, 2, 3a, 3b, 4, and 5 were categorized by eGFR (≥90, 60-90, 45-60, 30-45, 15-30, ≤15 mL/min/1.73 m^2^) [12]. The term “reduced kidney function” indicated eGFR < 60 mL/min/1.73 m^2^. The bias (mean difference) between each two equations was assessed by Bland-Altman plots. The differences in variables were analyzed appropriately by Student's *t*-test, the Mann-Whitney test, or the chi-square test. Kidney outcomes were compared using the log-rank test and demonstrated by the Kaplan-Meier curves method. The association between variables was analyzed by multivariable logistic regression. All analyses were conducted using SPSS software 22.0 and GraphPad Prism 7.0, and a two-sided *P* value of less than 0.05 was considered statistically significant.

## 3. Results

### 3.1. Baseline Demographic Clinical and Pathological Characteristics

A total of 308 patients with biopsy-proven DN were enrolled in the current study (Table 1). Among them, 69.5% of patients were male and the mean age was 51.4 ± 9.7 years old. The mean duration of diabetes was 97.8 ± 70.3 months. 44.8% and 86.0% of patients have diabetic retinopathy and hypertension, respectively. The mean serum creatinine and cystatin C were 130 ± 65 *μ*mol/L and 1.61 ± 0.60 mg/L, respectively. The median (interquartile range) initial proteinuria was 4.3 (2.0-7.8) g/24 hours. The thyroid levels were in normal range. The mean GFR was higher when estimated using the CKD-EPI-Cr (62.6 ± 28.7 mL/min/1.73 m^2^) than using CKD-EPI-CysC (50.3 ± 23.3 mL/min/1.73 m^2^) and CKD-EPI-Cr-CysC (54.6 ± 24.9 mL/min/1.73 m^2^). 81.5% of patients received RAAS inhibitors, 72.1% of patients received insulin therapy, and 57.5% of patients received statins.

All the patients in the study underwent kidney biopsy. Glomerular lesions were classified as follows. Seventeen patients had glomerular basement membrane thickening only and were classified as class I. 104 patients had mild or severe mesangial expansion, but without nodular sclerosis (Kimmelstiel-Wilson lesion), and were classified as class II. 140 patients who did not meet the criteria of class IV with at least one convincing Kimmelstiel-Wilson lesion were classified as class III. 47 patients with global glomerular sclerosis in ≥50% of glomeruli were classified as class IV. For the IFTA score, 10, 138, 127, and 33 patients were scored as 0, 1, 2, and 3, respectively. For interstitial inflammation, 20, 233, and 50 patients were scored as 0, 1, and 2, respectively. For arteriolar hyalinosis, 34, 162, and 112 patients were scored as 0, 1, and 2, respectively.

During a median follow-up period of 20 (13-33) months, a total of 131 (42.5%) patients reached ESKD.

### 3.2. CKD Stages Categorized Using Different Equations

**Figure 1:**
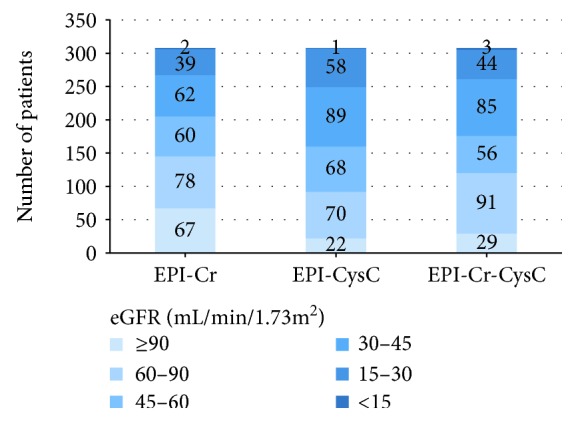
CKD stages categorized using different equations eGFR (mL/min/1.73m^2^). CKD stage 1: eGFR ≥ 90 mL/min/1.73m^2^; CKD stage 2: eGFR (60-90) mL/min/1.73m^2^; CKD stage 3a: eGFR (45-60) mL/min/1.73m^2^; CKD stage 3b: eGFR (30-45) mL/min/1.73m^2^; CKD stage 4: eGFR (15-30) mL/min/1.73m^2^; CKD stage 5: eGFR < 15 mL/min/1.73m^2^. More patients tend to be categorized into advanced CKD stages by eGFRCKD-EPI-CysC and eGFRCKD-EPI-Cr-CysC compared with eGFRCKD-EPI-Cr.

Figure 1 shows the proportion of CKD stages categorized using the different equations. Overall, more patients tend to be categorized into advanced CKD stages by eGFR_CKD-EPI-CysC_ and eGFR_CKD-EPI-Cr-CysC_ compared with eGFR_CKD-EPI-Cr_. Approximately half of patients (47%) were included in CKD 1 and CKD 2 stages when using equation of eGFR_CKD-EPI-Cr_, but only 30% of patients in CKD 1 and 2 stages when using equation of eGFR_CKD-EPI-CysC_. On the contrary, 62 patients were categorized into CKD 3b stage and 39 patients were in CKD 4 stage when using eGFR_CKD-EPI-Cr_, but 89 and 58 patients were in CKD 3b and 4 stages, respectively, when using eGFR_CKD-EPI-CysC_.

**Figure 2:**
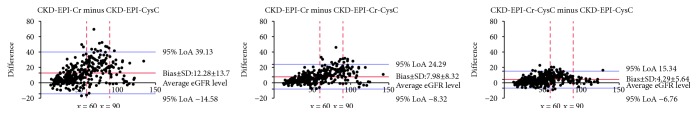
Bland-Altman plots of the difference between eGFRs. The limits of agreement (LoA) are defined as the mean difference ± 1.96 SD of differences. The black line and the blue lines indicate mean difference and 95% LoA, respectively. The red dashed lines indicate when average eGFR are 60 and 90 mL/min/1.73 m^2^. High disconcordance was observed between eGFR_CKD-EPI-Cr_ and eGFR_CKD-EPI-CysC_, especially when eGFR > 60 mL/min/1.73 m^2^. SD: standard deviation.

The Bland-Altman plot shown in Figure 2 revealed the bias between each two equations. Overall, high disconcordance was observed between eGFR_CKD-EPI-Cr_ and eGFR_CKD-EPI-CysC_, especially when eGFR ≥ 60 mL/min/1.73 m^2^. The mean bias of eGFR_CKD-EPI-Cr_ and eGFR_CKD-EPI-CysC_ was 12.28 ± 13.7 mL/min/1.73 m^2^, and upper 95% limit of agreement was 39.13 mL/min/1.73 m^2^, which were beyond accepted limit. The mean bias of eGFR_CKD-EPI-Cr_ and eGFR_CKD-EPI-Cr-CysC_ was 7.98 ± 8.32 mL/min/1.73 m^2^, and the mean bias of eGFR_CKD-EPI-Cr-CysC_ and eGFR_CKD-EPI-CysC_ was 4.29 ± 5.64 mL/min/1.73 m^2^.

### 3.3. Reclassification to Reduced Kidney Function by eGFR_CKD-EPI-CysC_ Equation

**Figure 3:**
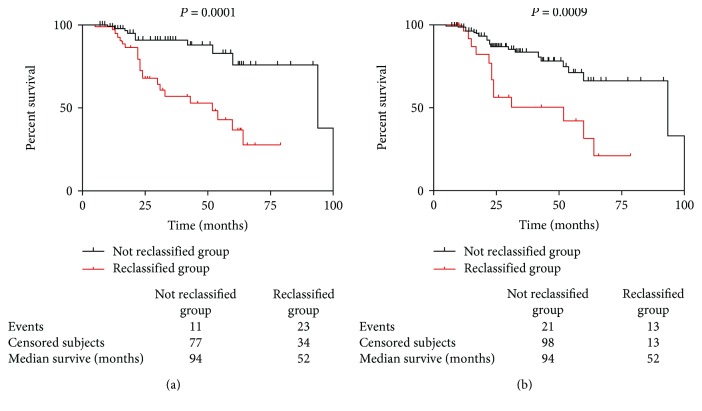
Kidney survival of patients reclassified in advanced CKD stages (3-5) by equations based on cystatin C. Comparison of kidney survival between the not reclassified group and reclassified group by eGFR_CKD-EPI-CysC_ (a) and eGFR_CKD-EPI-Cr-CysC_ (b). The kidney survival was significantly poorer by the log-rank test in the reclassified groups.

A total of 145 patients were with eGFR > 60 mL/min/1.73 m^2^ (CKD 1 and 2 stages) when using eGFR_CKD-EPI-Cr_ equation; however, 57 of them were reclassified into CKD 3-5 stages when using eGFR_CKD-EPI-CysC_ equation (Table 2). Compared with patients who were not reclassified (*n* = 88), the reclassified group presented significantly longer diabetic duration (106.8 ± 64.8 vs 83.4 ± 61.6 months), heavier proteinuria [4.2 (2.3-8.8) vs 2.4 (0.8-4.8) g/24 h, *P* = 0.001], higher level of cystatin C (1.45 ± 0.20 vs 1.02 ± 0.15 mg/L, *P* < 0.001), lower free triiodothyronine 3 [3.92 (3.46-4.35) vs 4.41 (3.92-5.10) pmol/L, *P* = 0.003], advanced glomerular classifications (*P* < 0.001), IFTA scores (*P* < 0.001), and interstitial inflammation (*P* < 0.001). In addition, during the follow-up, 11 (12.5%) patients reached the end point in the not reclassified group, while 21 (36.8%) patients reached the end point in the reclassified group; the kidney survival was significantly poorer by the log-rank test in the reclassified group (Figure 3(a)). Similarly, 26 patients were reclassified into CKD 3-5 stages using eGFR_CKD-EPI-Cr-CysC_ equation from patients with eGFR_CKD−EPI−Cr_ > 60 mL/min/1.73 m^2^. A significantly poorer kidney outcome was observed in patients who were reclassified by eGFR_CKD-EPI-Cr-CysC_ during the follow-up period (Figure 3(b)).

### 3.4. The Association between Serum Creatinine and Cystatin C and Pathological Lesions

We then investigated the association between serum creatinine and cystatin C and pathological lesions. For glomerular classification, we defined class of III and IV as advanced lesion, and for tubular and interstitial lesions, we defined scores of 2 and 3 of IFTA as advanced lesions. We adjusted essential clinical variables including gender, age, blood pressure, diabetic duration, triglyceride, total cholesterol, and proteinuria for multivariable logistic regression. As shown in Table 3, only gender (odds ratio (OR) 0.349, 95% confidence interval (CI) 0.174-0.700, *P* = 0.003) and cystatin C (OR 3.771, 95% CI 1.140-12.472, *P* = 0.030) were independently associated with advanced glomerular lesions. Serum creatinine (OR 1.004, 95% CI 0.993-1.015, *P* > 0.05) was not independently associated with advanced glomerular lesions. However, total cholesterol (OR 1.249, 95% CI 1.009-1.545, *P* = 0.041) and serum creatinine (OR 1.012, 95% CI 1.002-1.023, *P* = 0.024) were independently associated with advanced tubular and interstitial injury.

## 4. Discussion

Despite decades of research and heavy public health burden associated with DN, few new biomarkers have been applied to clinical practice in recent years [13]. Albuminuria and eGFR are still essential ones to monitor kidney function and guide management for patients with DN. However, the performance of GFR estimated by different equations is still under debate [14–16]. The current study showed the distribution of CKD stages categorized by different equations in patients with kidney-biopsy DN. 39% of patients with CKD 1 and 2 stages (by eGFR_CKD-EPI-Cr_) were reclassified into advanced CKD stages (by eGFR_CKD-EPI-CysC_) and they had longer diabetic duration, heavier proteinuria, advanced pathological lesions, and poorer prognosis. In addition, cystatin C, not creatinine, was independently associated with more severe glomerular classifications. Those findings suggest that equations incorporate cystatin C would improve the performance of detect glomerular lesions in the early stage in patients with DN.

Both serum creatinine and cystatin C are endogenous molecules. Serum creatinine is unstable and easily influenced by daily diet [10], secretion and reabsorption of tubular cells [17], and reduced muscle mass [18] which is common in patients with CKD [19]. Cystatin C is a low molecular basic protein, which is reabsorbed and catabolized by tubular cells completely. The serum concentrate is mainly affected by gender, obesity, diabetes, and hypertension [20, 21]. Criteria for selecting the optimal GFR estimating equation are accuracy, discrimination of kidney outcomes [22], and the population characteristic. Recently, substantial studies have evaluated the performance of different eGFR equations in the population of diabetes, CKD, or CKD with diabetes, but the conclusions remain largely controversial.

In the U.S. population of noninstitutionalized civilian, the prevalence of reduced kidney function was 6.5% when estimated using equations based on creatinine, compared with 8.7% when incorporated with cystatin C [23]. Similarly, a study enrolled 778 persons with diabetes detected the prevalence of reduced kidney function was 16.5% and 22.0% using eGFR_Cr_ and eGFR_CysC_, respectively. And patients with diabetes were more likely to be reclassified from preserved kidney function calculated by eGFR_Cr_ to reduced kidney function calculated by eGFR_CysC_ [20]. However, a study included 199 diabetic patients demonstrated that both eGFR_MDRD_ and eGFR_CKD-EPI-Cr_ equations underestimated measured GFR (>90 mL/min/1.73 m^2^) [24]. Moreover, long-term GFR decline was proved to be largely underestimated in a cross-sectional and longitudinal analysis [21]. In the current study, a biopsy-proven DN cohort, 53% of patients were with reduced kidney function using eGFR_CKD-EPI-Cr_ while 70% of which using eGFR_CKD-EPI-CysC_. Interestingly, patients who were reclassified by eGFR_CKD-EPI-CysC_ have significantly heavier proteinuria, advanced pathological lesions, and faster progression of kidney disease than not reclassified patients, which suggested that eGFR_CKD-EPI-CysC_ was more sensitive to detect kidney injury and predict kidney outcomes.

However, several studies questioned the improved performance of equations based on cystatin C. A latest study with 882 patients reported the misclassification was approximately 50% for creatinine-based equations and still 35% for cystatin C-based equations, and equations combined creatinine and cystatin C were not outperform equation only based on cystatin C [14]. In addition, eGFR_CysC_ failed to improve the area under the curve for the diagnosis of reduced kidney function in patients with diabetes [25]. In the current study, we found that cystatin C, not the creatinine, was independently associated with advanced glomerular lesions. The contradictory can be explained by the characteristics of subjects partially. First, different equations were compared in CKD cohorts which include various primary or secondary kidney diseases. Cystatin C may be influenced by different disease status; even in CKD with diabetes cohort, the nondiabetic kidney disease may confound results [26, 27]. Second, subjects in previous studies were characteristic with obese (BMI 28-31 kg/m^2^) and older (>55 years old). Higher BMI is associated with increased fat mass which is a primary determinant of cystatin C generation [26]. And the accurate of cystatin C is decreased with age [28]. In the current study, the impact of age (51 years old) and BMI (25.78 kg/m^2^) on the performance of eGFR_CysC_ is limited. Third, we aimed to explore the performance of equations to detect kidney injury, not the accuracy in estimating measured GFR.

These findings suggest that eGFR_CysC_ is more sensitive to detect kidney injury in the early stage. Therefore, eGFR_CysC_ should be considered, rather than eGFR_Cr_ alone, for clinical decision-making, especially when eGFR_Cr_ > 60 mL/min/1.73 m^2^.

There are several limitations of the current study that should be discussed. First, the sample size was limited due to that we only enrolled patients with biopsy-proven DN. Second, all the patients were ethnic Han in Southwest China; the performance of equations may be influenced by multiethnic setting such as muscle mass and meat intake. The results should be verified in more ethnic cohorts. Third, it was a retrospective cohort study; we did not have data of measured GFR to evaluate accuracy of different equations. Fourth, creatinine and cystatin C could be fluctuated; repeated measurements should be applied.

## 5. Conclusion

eGFR equations incorporating cystatin C is superior to eGFR based on creatine alone for detecting kidney injury in the early stage. The independent association between cystatin C and glomerular classifications might contribute to it.

## Figures and Tables

**Table 1:** Baseline demographic, clinical, and pathological characteristics.

Variables	Patients (*n* = 308)
Age (years)	51.4 ± 9.7
Gender (male)	214 (69.5)
Duration of diabetes (months)	97.8 ± 70.3
Diabetic retinopathy (%)	138 (44.8)
Body mass index (kg/m^2^)	25.78 ± 3.78
Hypertension (%)	265 (86.0)
Systolic blood pressure (mmHg)	145 ± 23
Diastolic blood pressure (mmHg)	86 ± 13
HbA1C (%)	7.3 (6.3-8.5)
Initial proteinuria (g/24 h)	4.3 (2.0-7.8)
Serum creatinine (*μ*mol/L)	130 ± 65
Cystatin C (mg/L)	1.61 ± 0.60
Triglyceride (mmol/L)	2.19 ± 1.80
Total cholesterol (mmol/L)	5.22 ± 1.60
Thyroid-stimulating hormone (mU/L)	3.15 (1.96-5.60) (*n* = 155)
Free triiodothyronine 3 (pmol/L)	3.92 (3.39-4.41) (*n* = 151)
Free triiodothyronine 4 (pmol/L)	14.95 (13.17-16.61) (*n* = 154)
eGFR_CKD-EPI-Cr_	62.6 ± 28.7
eGFR_CKD-EPI-CysC_	50.3 ± 23.3
eGFR_CKD-EPI-Cr-CysC_	54.6 ± 24.9
RAAS inhibitors (%)	251 (81.5)
Insulin therapy (%)	222 (72.1)
Statins (%)	177 (57.5)
Glomerular class	
I	17
IIa	75
IIb	29
III	140
IV	47
Interstitial fibrosis and tubular atrophy	
0	10
1	138
2	127
3	33
Interstitial inflammation	
0	20
1	233
2	55
Arteriolar hyalinosis	
0	34
1	162
2	112
Reach end-stage kidney disease	131
Median kidney survival time (months)	20 (13-33)

Data are presented as the mean ± standard and the median with range or counts and percentages.

**Table 2:** Clinical and pathological characteristics of the not/reclassified group.

Variables	Not reclassified group (*n* = 88)	Reclassified group (*n* = 57)	*P* value
eGFR_CKD-EPI-Cr_	96.5 ± 17.0	76.1 ± 11.5	<0.001
eGFR_CKD-EPI-CysC_	79.9 ± 17.1	49.3 ± 7.2	<0.001
Age (years)	49.4 ± 10.8	51.3 ± 8.7	NS
Gender (male) (%)	62 (70.5)	38 (66.7)	NS
Duration of diabetes (months)	83.4 ± 61.6	106.8 ± 64.8	0.033
Diabetic retinopathy (%)	28 (32.6)	22 (40.0)	NS
Body mass index (kg/m^2^)	26.24 ± 3.35	25.67 ± 4.59	NS
Systolic blood pressure (mmHg)	138 ± 20	144 ± 22	NS
Diastolic blood pressure (mmHg)	86 ± 14	85 ± 13	NS
HbA1C (%)	7.8 (6.8-9.4)	7.5 (6.3-10.2)	NS
Initial proteinuria (g/24 h)	2.4 (0.8-4.8)	4.2 (2.3-8.8)	0.001
Serum creatinine (*μ*mol/L)	74 ± 18	93 ± 19	<0.001
Cystatin C (mg/L)	1.02 ± 0.15	1.45 ± 0.20	<0.001
Triglyceride (mmol/L)	2.39 ± 2.17	2.03 ± 1.61	NS
Total cholesterol (mmol/L)	4.89 ± 1.36	5.36 ± 1.61	NS
Thyroid-stimulating hormone (mU/L)	2.93 (1.79-4.09) (*n* = 43)	3.86 (2.61-6.58) (*n* = 29)	NS
Free triiodothyronine 3 (pmol/L)	4.41 (3.92-5.10) (*n* = 42)	3.92 (3.46-4.35) (*n* = 29)	0.003
Free triiodothyronine 4 (pmol/L)	15.87 (14.65-17.26) (*n* = 42)	14.54 (12.50-16.66) (*n* = 29)	NS

Pathological lesions			
Glomerular classification			<0.001
I	14	1	
IIa	39	14	
IIb	6	7	
III	26	29	
IV	3	6	
IFTA scores			<0.001
0	8	2	
1	62	27	
2	18	25	
3	0	3	
Interstitial inflammation			<0.001
0	17	2	
1	70	45	
2	1	10	
Arteriolar hyalinosis			NS
0	18	10	
1	47	28	
2	23	19	
Follow-up period (months)	32 ± 22	30 ± 18	NS
Progressed to ESKD (%)	11 (12.5)	21 (36.8)	<0.001

Patients in the reclassified group had significantly longer diabetic duration, heavier proteinuria, and advanced pathological lesions. Data are presented as the mean ± standard and the median with range or counts and percentages. IFTA: interstitial fibrosis and tubular atrophy; NS: not significant. A two-tailed *P* < 0.05 was considered statistically significant.

**Table 3:** Multivariable logistic regression of advanced glomerular classification and IFTA.

Variables	Glomerular classification of III and IV			IFTA of 2 and 3 scores		
	Odds ratio	95% CI	*P*	Odds ratio	95% CI	*P*
Gender (male)	0.349	0.174-0.700	0.003	1.037	0.538-2.000	NS
Age	0.985	0.955-1.015	NS	0.985	0.956-1.015	NS
Systolic blood pressure	1.008	0.991-1.025	NS	1.004	0.988-1.020	NS
Diastolic blood pressure	0.995	0.966-1.025	NS	1.015	0.987-1.043	NS
Diabetic duration	1.003	0.999-1.008	NS	1.001	0.997-1.005	NS
Triglyceride	0.905	0.769-1.063	NS	0.941	0.803-1.104	NS
Total cholesterol	1.022	0.821-1.271	NS	1.249	1.009-1.545	0.041
Proteinuria	1.059	0.979-1.146	NS	1.018	0.949-1.092	NS
Serum creatinine	1.004	0.993-1.015	NS	1.012	1.002-1.023	0.024
Cystatin C	3.771	1.140-12.472	0.030	1.680	0.576-4.902	NS

Gender and cystatin C were independently associated with advanced glomerular classifications (III and IV stages); total cholesterol and serum creatinine were independently associated with higher IFTA scores. IFTA: interstitial fibrosis and tubular atrophy; CI: confidence interval; NS: not significant. A two-tailed *P* < 0.05 was considered statistically significant.

## Data Availability

Original data can be provided if editors require.
